# Single-cell sequencing reveals an important role of SPP1 and microglial activation in age-related macular degeneration

**DOI:** 10.3389/fncel.2023.1322451

**Published:** 2024-01-08

**Authors:** Shizhen Lei, Mang Hu, Zhongtao Wei

**Affiliations:** ^1^Department of Ophthalmology, Wuhan No. 1 Hospital, Tongji Medical College, Huazhong University of Science and Technology, Wuhan, Hubei, China; ^2^Wuhan Fourth Hospital, Wuhan, Hubei, China

**Keywords:** age-related macular degeneration, senescence, SPP1, microglia, single-cell RNA sequencing

## Abstract

**Purpose:**

To investigate the role of senescence-related cytokines (SRCs) in the pathophysiology of age-related macular degeneration (AMD).

**Design:**

The whole study is based on single-cell and bulk tissue transcriptomic analysis of the human neuroretinas with or without AMD. The transcriptomic data of human neuroretinas was obtained from Gene-Expression Omnibus (GEO) database.

**Methods:**

For single-cell transcriptomic analysis, the gene expression matrix goes through quality control (QC) filtering, being normalized, scaled and integrated for downstream analysis. The further analyses were performed using Seurat R package and CellChat R package. After cell type annotation, the expression of phenotype and functional markers of microglia was investigated and cell-cell communication analysis was performed. For bulk tissue transcriptomic analysis, GSE29801 dataset contains the transcriptomic data of human macular neuroretina (*n* = 118) from control group and AMD patients. The expression of SPP1 in control and AMD subtypes were compared by Student’s *t*-test. In addition, the AMD macular neuroretina were classified into SPP1-low and SPP1-high groups according to the expression level of SPP1. The differentially expressed genes between these two groups were subsequently identified and the pathway enrichment analysis for these genes was further conducted.

**Results:**

Secreted phosphoprotein 1, as an SRC, was revealed to be highly expressed in microglia of AMD neuroretina and the SPP1-receptor signaling was highly activated in AMD neuroretina. In addition, SPP1 signaling was associated with the pro-inflammatory phenotype and phagocytic state of microglia. SPP1 expression was elevated in macular neuroretina with late dry and wet AMD and the inflammatory pathways were found to be activated in SPP1-high AMD macular neuroretina.

**Conclusion:**

Our findings indicated that SPP1 and microglial activation might play an important role in the pathophysiology of AMD. Therefore, SPP1 might serve as a potential therapeutic target for AMD. More *in vitro* and *in vivo* studies are required to confirm the results and the therapeutic effect of SPP1-targeting strategy.

## 1 Introduction

Worldwide, the growing incidence of age-related diseases have imposed a major socioeconomic and public health challenge. Aging is a major risk factor for several vision-threatening diseases affecting the retina, including age-related macular degeneration (AMD). AMD is a widely diagnosed neurodegenerative disease, which affects the macula (the most important part of the retina) (Wong et al., 2014) and has been associated with visual impairment and considerable health burden (Taylor et al., 2016; Zhu et al., 2019). The number of individuals affected by AMD is keeping increasing (Congdon et al., 2004; Wong et al., 2014). Despite recent efforts and advances, the mechanisms underlying the AMD pathophysiology is still not fully understood.

Intravitreal injection of anti-vascular endothelial growth factor (VEGF), including ranibizumab (Blodi et al., 2023) and aflibercept (Wykoff et al., 2023), have been used to slow the progression of the neovascular/wet subtype (nAMD/wAMD). However, since nAMD patients may not show response to anti-VEGF treatment (Tenbrock et al., 2022) and there is no effective treatment available for atrophic/dry AMD, there is still an urgent need for further investigation of the pathogenesis and treatment options.

Cellular senescence has been suggested to play a critical role (Donoso et al., 2010; Kauppinen et al., 2016) in AMD initiation and development. As a hallmark of aging, cellular senescence is a significant contributor to aging and age-related diseases including AMD (Holloway et al., 2023). Notably, Saul et al. (2022) has identified a gene set (senescence-related genes, SRGs) for predicting senescence-associated pathways across tissues, which has been used and cited by many researches about age-related diseases (Doolittle et al., 2023; Farr, 2023; Yu et al., 2023). Importantly, the senescence-related cytokines (SRCs) were the key part of this senescence-related gene set.

In this study, we investigated the expression and potential role of SRCs in the pathophysiology of AMD. As a result, single-cell and bulk tissue transcriptomic analysis showed that SPP1 was the most important SRC in the development of AMD and revealed the vital role of SPP1 signaling and microglial activation in AMD development.

## 2 Materials and methods

### 2.1 Single-cell transcriptomic analysis

The list of 35 SRCs was obtained from Saul et al. (2022) (Supplementary Table 1). We obtained single-cell RNA (scRNA) sequencing data of AMD neuroretina from GSE137537 dataset in Gene-Expression Omnibus (GEO) database. In this dataset, isolated and sequenced neuroretinal cells from neuroretinal suspensions of three postmortem human eyes diagnosed with AMD. Following quality control (QC) filtering, the gene expression matrix was normalized, scaled and integrated for downstream analysis (Butler et al., 2018). The further analyses were performed using Seurat R package (version 4.0) (Gribov et al., 2010) and CellChat R package (version 1.6.1) (Liu et al., 2022). To assign cluster identities, the markers of clusters were identified by using the FindAllMarkers function in Seurat. Clustering and uniform manifold approximation and projection (UMAP) dimensional reduction were performed and cell annotation markers provided by the authors of the dataset (Menon et al., 2019) were used to identify the cell types in the neuroretina tissue including: Amacrine cell (GAD1, C1QL2), Bipolar cell (CAMK2B, GRM6, TMEM215, TRPM1), Cone (GNAT2, OPN1SW, OPN1MW, OPN1LW), Endothelial cell (CD34, CDH5, RGS5, ADAMTS9, DLL4, FLT1, KDR, VWF), Horizontal cell (ONECUT1, ONECUT2, LHX1), Microglia (C1QA, TMEM119, AIF1, CD163), Müller glia and Astrocyte (GLUL, CLU, APOE), Retinal ganglion cell (NEFM, SLC17A6), Rod (PDE6A, PPEF2, NR2E3) (Supplementary Table 2). Visualization of gene expression was generated using the functions in the Seurat package.

To explore the cell-cell communications between retinal cell types from scRNA sequencing data, CellPhoneDB database^1^ was used to perform ligand-receptor pairing analysis (Efremova et al., 2020), which contains summarized set of protein-protein interactions (PPI). CellPhoneDB statistical analysis was performed using the “secreted signaling” set (cytokines and chemokines) between all cell populations.

### 2.2 Bulk tissue transcriptomic analysis

The bulk tissue RNA sequencing data of human macular neuroretina samples were also obtained from GEO database. GSE29801 reported the bulk tissue transcriptomic atlas of human macular neuroretina (*n* = 118) from control group and AMD patients (Newman et al., 2012). The AMD macular neuroretinas were classified into three subtypes, including early AMD, late dry AMD (geographic atrophy, GA) and wet/neovascular AMD, according to Age Related Eye Disease Studies (AREDS).

The expression of SPP1 in control and AMD subtypes were compared. In addition, the AMD macular neuroretinas were classified into SPP1-low and SPP1-high groups according to the expression level of SPP1. The differentially expressed genes (DEGs) between these two groups were subsequently identified. The criteria for DEGs were adjusted *P* < 0.05 and | log (FC) | > 1. The pathway enrichment analysis for these DEGs was performed via Metascape database.^2^

### 2.3 Statistical analysis

R software (version 4.0.1) and GraphPad Prism (version 8.0) was used for statistical analysis. Values between two groups were compared using Student’s *t*-test or Wilcoxon rank-sum test. All *P-*values were two-sided and the threshold for significance was set as *P* < 0.05.

## 3 Results

### 3.1 SRCs expression and cell-cell communications in AMD retina

**FIGURE 1:**
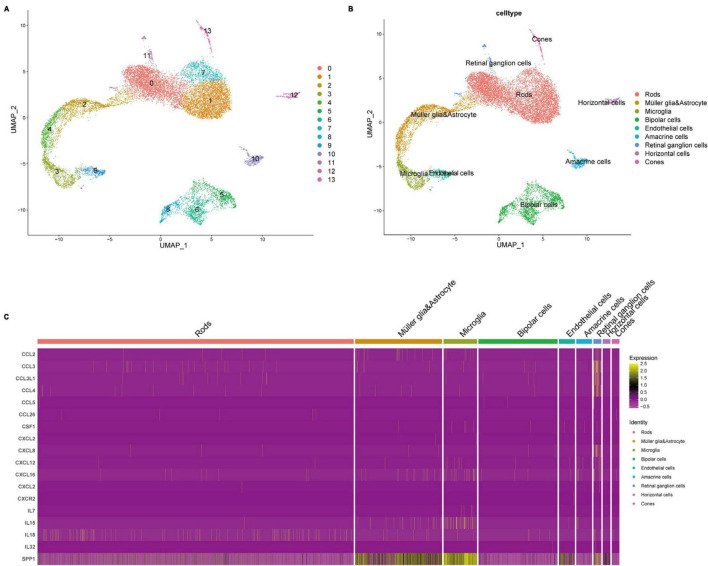
Cell atlas and gene expression analysis of SRCs in AMD retinas. **(A,B)** The clustering and cell annotation of the single-cell transcriptomic data of retinas with AMD; **(C)** Heatmap of SRCs expression in different retinal cell types. SRC, senescence-related cytokine; AMD, age-related macular degeneration.

**FIGURE 2:**
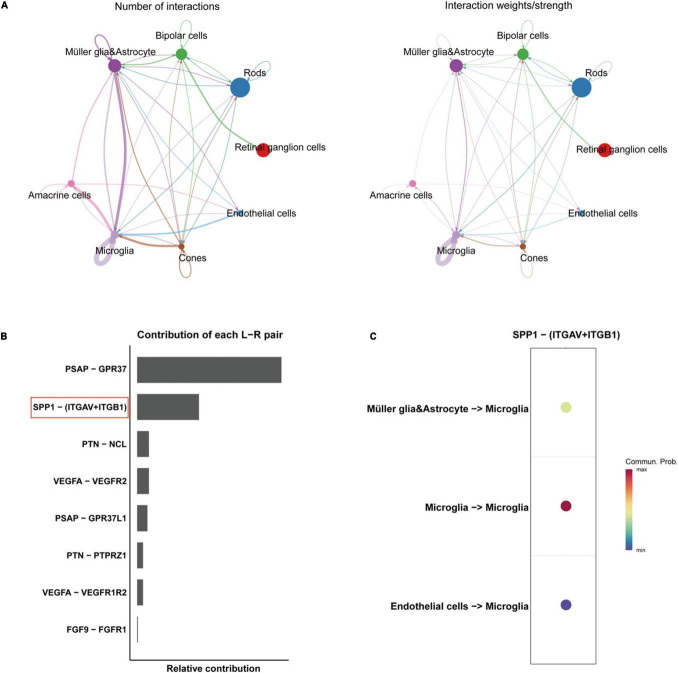
Cell-cell communications in AMD retinas. **(A)** The number and weights/strength of cell-cell interactions by secreted signaling in AMD retina; **(B)** The contribution of each ligand-receptor (L-R) pair in the cell-cell communication network. **(C)** The directions of SPP1-receptor signaling AMD retina. AMD, age-related macular degeneration.

**FIGURE 3:**
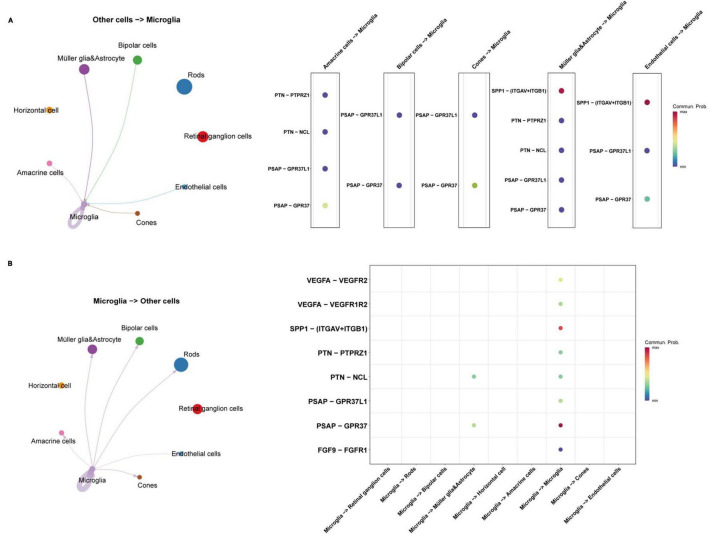
The communications between microglia and other retinal cell types in AMD retina. **(A)** The interactions from other retinal cells to microglia; **(B)** The interactions from microglia to other retinal cells. AMD, age-related macular degeneration.

Through quality control (QC) filtering, normalization and cell type annotation, 9 types of neuroretinal cells were identified: 2,832 Retinal ganglion cells (RGCs); 4,741 Rods; 1,989 Bipolar cells; 2,548 Macroglia (Müller glia and Astrocyte); 986 Horizontal cell; 535 Amacrine cells; 869 Microglia; 219 Cones; and 245 Endothelial cells (Figures 1A, B). The expression of SRCs was investigated and single-cell transcriptomic analysis showed that SPP1 was a highly expressed SRC in AMD neuroretina, which was mainly expressed in Microglia, Müller glia and Astrocyte, and Endothelial cells in AMD neuroretina (Figure 1C). Notably, cell-cell communication analysis (secreted signaling) showed that neuroretinal cells communicated frequently with each other (Figure 2A) and the SPP1-(ITGAV + ITGB1) signaling was highly activated in AMD neuroretina (Figure 2B). ITGAV (Integrin Subunit Alpha V) and ITGB1 (Integrin Subunit Beta 1) belong to integrin family and can act as the receptors of SPP1 (Kiefer et al., 1989; Wai and Kuo, 2004). In addition, SPP1-(ITGAV + ITGB1) signaling existed in Müller glia and Astrocyte to Microglia, Endothelial cells to Microglia, and Microglia to Microglia (Figure 2C). Therefore, we investigated all the secreted communications between microglia and other neuroretinal cells and the results indicated that SPP1-(ITGAV + ITGB1) signaling stimulated and activated microglia through paracrine and autocrine ways (Figures 3A, B).

**FIGURE 4:**
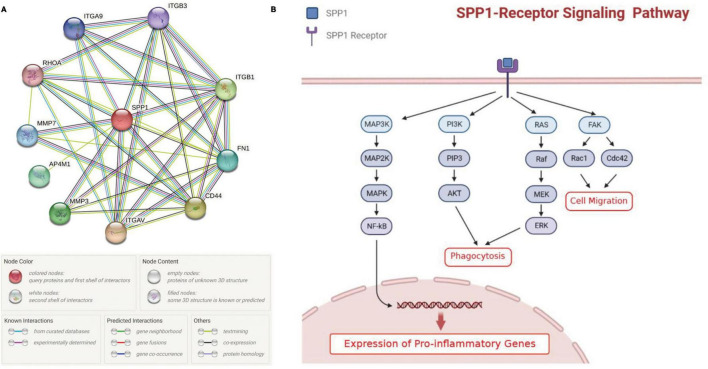
The effect of SPP1 signaling on microglia. **(A)** the PPI network of SPP1 and its receptors or non-receptor factors; **(B)** The summary of SPP1 signaling pathways in retinal microglia identified in previous studies. PPI, protein-protein interaction; AMD, age-related macular degeneration.

The protein-protein interaction (PPI) network from String database showed that SPP1 could interact with multiple receptors including CD44, ITGAV, ITGA9, ITGB1, and ITGB3 and non-receptor factors such as MMP3 and MMP7 (Figure 4A). Previous publications suggested that SPP1-receptor signaling in microglia could lead to the elevated expression of proinflammatory genes, promote phagocytosis and cell migration (Figure 4B; Rosmus et al., 2022).

**FIGURE 5:**
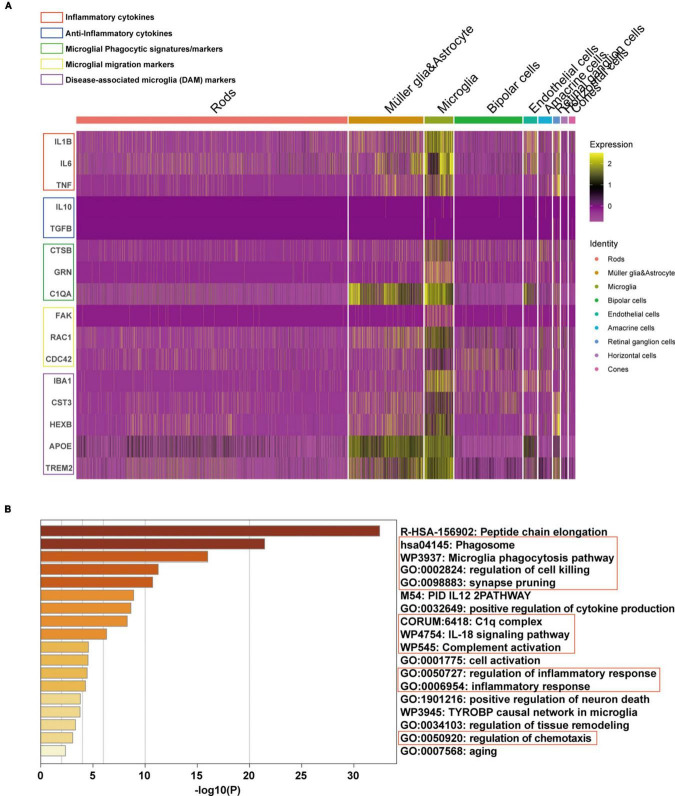
Phenotype and functional state of microglia in microglia of AMD neuroretina. **(A)** The expression of phenotype and functional markers in microglia of AMD neuroretina; **(B)** Up-regulated pathways in retinal microglia under the condition of AMD. AMD, age-related macular degeneration.

Subsequently, the expression of phenotype and functional markers of microglia in AMD neuroretina was investigated. The results showed that inflammatory cytokines (including IL1B, IL6, and TNF) (Fang et al., 2023), microglial phagocytic markers (CTSB, GRN, and C1QA) (De Schepper et al., 2023), cell migration signatures (FAK, RAC1, and CDC42) (Rosmus et al., 2022), and classic disease-associated microglia (DAM) markers (IBA1, CST3, HEXB, APOE, and TREM2) (Deczkowska et al., 2018) were upregulated in microglia, while the anti-inflammatory cytokines (IL10 and TGFB) (Fang et al., 2023) were at low expression levels in microglia (Figure 5A). Pathway enrichment analysis was then performed and the mainly upregulated pathways in microglia of AMD neuroretina included phagocytosis-related pathways (Phagosome, Microglia phagocytosis pathway, Synapse pruning, Regulation of cell killing, and Positive regulation of neuron death), complete activation-associated pathways (C1q complex and Complete activation), inflammation-related pathways (IL-18 signaling pathway and Regulation of inflammatory response) and Regulation of chemotaxis pathway (Figure 5B).

### 3.2 SPP1 expression and associated pathways in AMD macular neuroretina

**FIGURE 6:**
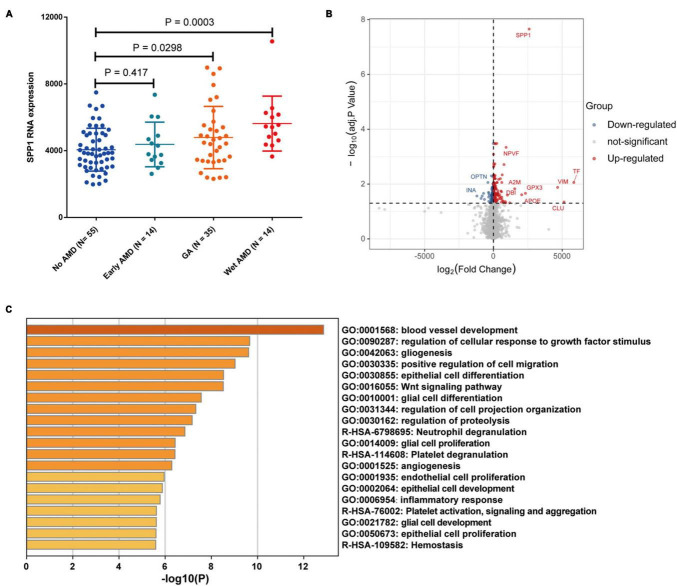
The SPP1 expression in AMD macular neuroretina. **(A)** The comparison of SPP1 expression in macular neuroretina with or without different AMD subtypes; **(B)** The DEGs between SPP1-low and SPP1-high macular neuroretina with AMD; **(C)** The up-regulated pathways in SPP1-high macular neuroretina. AMD, age-related macular degeneration.

Bulk transcriptomic analysis showed that SPP1 expression was significantly elevated in macular neuroretina with GA and neovascular/wet AMD (*P* < 0.05), compared to normal ones (Figure 6A). The DEGs between SPP1-low and SPP1-high AMD neuroretina were identified (Figure 6B).

The upregulated pathways in SPP1-high AMD macular neuroretina included angiogenesis-related pathways (blood vessel development and angiogenesis), glia-activation pathways (gliogenesis, glial cell proliferation, glial cell differentiation, and glial cell development), Inflammatory response pathway, and Endothelial cell proliferation pathway (Figure 6C).

## 4 Discussion

There are several stages of AMD, i.e., the early stage and the late or advanced stages. The important characteristic of the former is the occurrence of drusen, which is the extracellular accumulation of lipid, minerals and proteins (Fleckenstein et al., 2021). The late or advanced stages of this disease can be classified into atrophic/dry and neovascular/wet forms (nAMD). The atrophic/dry form of AMD could end as the extensive atrophy of whole layers of the retina, called geographic atrophy (GA). While nAMD is characterized by the formation of choroidal neovascularization (CNV) (Tenbrock et al., 2022). For atrophic/dry AMD, there is no effective treatment available. For nAMD, the anti-VEGF therapy is also limited as patients with nAMD may not show response to this treatment. Therefore, it is urgently needed to further investigate the pathophysiology and develop novel therapeutic targets.

Secreted phosphoprotein 1 (SPP1)/Osteopontin (OPN) is a protein with multiple functions and widely expressed in tissues of human body (Cappellano et al., 2021). Recently, the upregulation of SPP1 has been revealed in various autoimmune diseases [including systemic lupus erythematosus (Wong et al., 2005)], inflammatory diseases [including Crohn’s disease (Sato et al., 2005) and multiple sclerosis (MS) (Chabas et al., 2001)], and neurodegenerative diseases (NDDs) such as Alzheimer’s disease (AD) (Wung et al., 2007; Comi et al., 2010). In addition, AMD is also one type of NDDs. Therefore, the role of SPP1 in pathophysiology of AMD is worthy of further investigation.

Currently, microglia, as one type of neuroglial cells, have been found to closely interact with other cells in both central and peripheral nervous system, which is important for the maintenance of nervous tissues. Notably, it has been suggested that the migration of activated microglia to the ongoing retinal lesion and their phenotype transformation are the hallmarks of AMD pathogenesis (Zhao et al., 2023). In addition, the crosstalk between microglia, Müller glia and astrocyte plays an important role in AMD pathology process (Cuenca et al., 2014; Zhao et al., 2023). In this study, cell-cell communication analysis showed that Müller glia and astrocyte interact with microglia via secreted SPP1. Moreover, the expression of cell-migration signatures (FAK, RAC1, and CDC42) (Rosmus et al., 2022) was elevated in microglia of AMD neuroretina. The glial activation pathways were also activated in SPP1-high AMD macular neuroretina. These results indicated the important role of SPP1 in this AMD-promoting crosstalk between glial cells.

Activated microglia, similar to peripheral macrophages, have two primary polarization phenotypes: M1 (classical activation) and M2 (alternative activation) (Wolf et al., 2017). M1-phenotype microglia mainly play a pro-inflammatory role and promote the synthesis of inflammatory mediators such as tumor necrosis factor-alpha (TNF-α), interleukin-6 (IL-6), and interleukin-1β (IL-1β), which can promote chronic neuroinflammation, phagocytosis, and neurodegeneration and inhibit regeneration (Orihuela et al., 2016). Therefore, M1 microglia often have obvious neurotoxic effects (Kwon and Koh, 2020). In contrast, the M2-phenotype microglia are alternatively activated microglia, which release neuroprotective and anti-inflammatory cytokines, such as interleukin-10 (IL-10) and transforming growth factor-β (TGF-β), to exert anti-inflammatory effects, promote wound healing, neuroprotection, and tissue repair (Liu et al., 2020). Therefore, M2 microglia often have neuroprotective effects (Tang and Le, 2016). In this study, single-cell sequencing analysis showed that inflammatory cytokines were upregulated and anti-inflammatory cytokines were downregulated in retinal microglia of AMD neuroretina, which indicated the neurotoxic M1-phenotype, instead of neuroprotective M2-phenotype, of microglia in AMD development and highlighted the important role of activated microglia in the formation of neuroinflammation in AMD.

Phagocytosis of microglia has been implicated in NDDs. For example, in AD, the phagocytic function of microglia plays a dual role. On the one hand, it can promote the clearance of toxic Aβ peptides (Zeng et al., 2023); on the other hand, the phagocytic state in microglia induces synaptic engulfment and subsequent synaptic loss and neuron death in AD (De Schepper et al., 2023). Notably, the loss of photoreceptors and synapse elimination has been observed in AMD (Yednock et al., 2022). In AMD, the injury of blood-retinal barrier allows leakage of serum proteins, including C1q and other complement components, into the retina from the underlying choriocapillaris (Katschke et al., 2018). Local complement activation leads to the recruitment of microglia into the lesion, which then produce additional C1q and other complement components, prune complement-coated synapses away from neurons, depriving neurons of trophic support, and cause neuroinflammation that adds to neuronal damage and loss (Stephan et al., 2012). In this study, we noticed the high expression of C1QA and other phagocytic markers including CTSB and GRN (De Schepper et al., 2023) in microglia of AMD neuroretina. The phagocytosis, cell killing and chemotaxis pathways were also found to be activated in microglia of AMD neuroretina. Interestingly, De Schepper et al. (2023) reported that SPP1 could induce the phagocytic state and the neuron damage effects in microglia in AD. Publications also suggested that SPP1 could promote phagocytosis and cell migration of microglia (Rosmus et al., 2022). These results indicated that SPP1 may promote the development of AMD by activating and inducing the phagocytic state of retinal microglia, thereby causing damage to photoreceptors and corresponding visual impairment.

The important role of the special status of microglia, called disease-associated microglia (DAMs), in NDDs has been revealed by studies (Prinz et al., 2019). DAMs have unique patterns of gene expression and are associated with neuroinflammatory and neurodegenerative conditions (Keren-Shaul et al., 2017; Jordão et al., 2019; Masuda et al., 2019; O’Koren et al., 2019; Wieghofer et al., 2021). DAMs are characterized by expressing typical microglial markers, IBA1, CST3, and HEXB (Butovsky et al., 2014), which were observed to be highly expressed in microglia of AMD neuroretina in this study. DAMs further display upregulation of genes involved in lysosomal, phagocytic, and lipid metabolism pathways, such as APOE and TREM2, which are known AD risk factors (Lambert et al., 2013). Moreover, previous studies have suggested the potential synergy between complete system and TREM2-APOE signaling in inducing synaptic pruning of microglia and neuron loss in AD (Shi and Holtzman, 2018; Linnartz-Gerlach et al., 2019). Interestingly, results in this study found the activation of both complete system and TREM2-APOE signaling in microglia of AMD neuroretina, indicating the synergy between these two systems also existed in AMD development. Notably, high expression of SPP1 has been identified as an important characteristic of the DAMs in AD brain (Keren-Shaul et al., 2017). Therefore, SPP1 may stimulate microglia and contribute to synaptic loss and death of photoreceptors via these two mechanisms.

Moreover, the angiogenesis-related pathways were found to be activated in SPP1-high AMD macular neuroretina. In addition, SPP1 expression was also elevated in macular neuroretina with neovascular/wet AMD. Interestingly, a previous study has revealed that the SPP1 expression is upregulated in laser-induced CNV and microglia in CNV membranes from patients with neovascular AMD (Schlecht et al., 2020). It has also been suggested that SPP1 may promote angiogenesis by stimulating integrin receptor signaling and then PI3K/AKT- and ERK1/2 signaling pathways in endothelial cells (Dai et al., 2009). Of note, a recent study demonstrated that intravitreal injection of an SPP1-blocking antibody could reduce CNV sizes in mice (Beguier et al., 2020).

**FIGURE 7:**
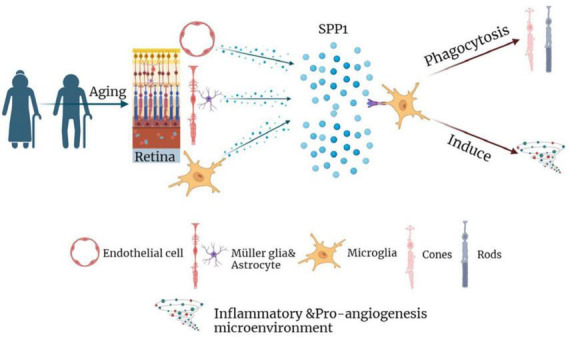
The diagram summarizing the role of SPP1 and microglial activation in the pathophysiology of AMD. AMD, age-related macular degeneration.

Collectively, the results indicated that SPP1 might activate retinal microglia, thereby inducing the synaptic loss and subsequent death of photoreceptors, neuroinflammation, and the formation of pro-angiogenesis microenvironment during AMD development (Figure 7). Therefore, SPP1 might serve as a potential therapeutic target for AMD. Still, further *in vitro* and *in vivo* studies are required to confirm the results and the therapeutic effect of SPP1-based strategy.

## Data availability statement

The original contributions presented in this study are included in this article/Supplementary material, further inquiries can be directed to the corresponding author.

## Ethics statement

This study used only publicly available, deidentified data from previously published works, making it exempt according to the Wuhan Fourth Hospital institutional review board. Our research adhered to the tenets of the Declaration of Helsinki.

## Author contributions

SL: Conceptualization, Data curation, Formal analysis, Investigation, Methodology, Project administration, Visualization, Writing—original draft. MH: Conceptualization, Data curation, Formal analysis, Writing—original draft. ZW: Conceptualization, Methodology, Supervision, Writing—review and editing.

## Footnotes

1
https://www.cellphonedb.org/

2
https://metascape.org/gp/index.html
