# Supplementary material for: Single-cell sequencing reveals an important role of SPP1 and microglial activation in age-related macular degeneration
Source: Front Cell Neurosci. 2024 Jan 8;17:1322451. doi: 10.3389/fncel.2023.1322451 (PMC10801008; doi:10.3389/fncel.2023.1322451)
Supplement: Supplementary file 1 [file Table_1.PDF]

SUPPLEMENTAL MATERIAL

Single-cell sequencing reveals an important role of SPP1 and microglial activation in age-related macular degeneration

Shizhen Lei, [1,†] Mang Hu, [1,†] Zhongtao Wei, [2,*]

SUPPLEMENTAL TABLE

Supplemental Table 1. The 35 senescence-related cytokines from Saul et al.

Gene symbol	Category
CCL1	Cytokine/Chemokine
CCL13	Cytokine/Chemokine
CCL16	Cytokine/Chemokine
CCL2	Cytokine/Chemokine

CCL20	Cytokine/Chemokine
CCL24	Cytokine/Chemokine
CCL26	Cytokine/Chemokine
CCL3	Cytokine/Chemokine
CCL3L1	Cytokine/Chemokine
CCL4	Cytokine/Chemokine
CCL5	Cytokine/Chemokine
CCL7	Cytokine/Chemokine
CCL8	Cytokine/Chemokine
CSF1	Cytokine/Chemokine
CSF2	Cytokine/Chemokine
CXCL1	Cytokine/Chemokine
CXCL10	Cytokine/Chemokine
CXCL12	Cytokine/Chemokine
CXCL16	Cytokine/Chemokine
CXCL2	Cytokine/Chemokine
CXCL3	Cytokine/Chemokine
CXCL8	Cytokine/Chemokine
CXCR2	Cytokine/Chemokine
IL10	Cytokine/Chemokine
IL13	Cytokine/Chemokine
IL15	Cytokine/Chemokine
IL18	Cytokine/Chemokine
IL1A	Cytokine/Chemokine
IL1B	Cytokine/Chemokine
IL2	Cytokine/Chemokine
IL32	Cytokine/Chemokine
IL6	Cytokine/Chemokine
IL7	Cytokine/Chemokine
SPP1	Cytokine/Chemokine
TNF	Cytokine/Chemokine

Supplemental Table 2. Specific markers used for cell type annotation.

Cell name	Cell marker

Amacrine cell	GAD1, C1QL2
Bipolar cell	CAMK2B, GRM6, TMEM215, TRPM1
Cone	GNAT2, OPN1SW, OPN1MW, OPN1LW
Endothelial cell	CD34, CDH5, RGS5, ADAMTS9, DLL4, FLT1, KDR, VWF
Horizontal cell	ONECUT1, ONECUT2, LHX1
Microglia	C1QA, TMEM119, AIF1, CD163
Müller glia & Astrocyte	GLUL, CLU, APOE
Retinal ganglion cell	NEFM, SLC17A6
Rod cell	PDE6A, PPEF2, NR2E3
